# Tracheal Penetration and Tracheoesophageal Fistula Caused by an Esophageal Self-Expanding Metallic Stent

**DOI:** 10.1155/2014/567582

**Published:** 2014-09-03

**Authors:** Karan Madan, Arun Venuthurimilli, Vineet Ahuja, Vijay Hadda, Anant Mohan, Randeep Guleria

**Affiliations:** ^1^Department of Pulmonary Medicine & Sleep Disorders, All India Institute of Medical Sciences (AIIMS), Ansari Nagar, New Delhi 110029, India; ^2^Department of Gastrointestinal Surgery, All India Institute of Medical Sciences (AIIMS), Ansari Nagar, New Delhi 110029, India; ^3^Department of Gastroenterology, All India Institute of Medical Sciences (AIIMS), Ansari Nagar, New Delhi 110029, India

## Abstract

Tracheal penetration of esophageal self-expanding metallic stents (SEMS) with/without tracheoesophageal fistula (TEF) formation is a rare occurrence. We report the case of a 66-year-old female patient with advanced esophageal squamous cell carcinoma who had undergone palliative esophageal stenting on three occasions for recurrent esophageal stent obstruction. On evaluation of symptoms of breathing difficulty and aspiration following third esophageal stent placement, tracheal erosion and TEF formation due to the tracheal penetration by esophageal stent were diagnosed. The patient was successfully managed by covered tracheal SEMS placement under flexible bronchoscopy.

## 1. Introduction

Esophageal SEMS placement has evolved into a first line approach for the palliative relief of dysphagia for patients with advanced esophageal cancer [1]. The success rate for esophageal stent placement (performed under fluoroscopic or endoscopic guidance) is high and early complications related to stent placement are uncommon and usually self-limited. Indications for esophageal SEMS (covered or uncovered stents) placement have gradually evolved and include malignant esophageal obstructions, malignant esophageal perforations, and esophageal airway fistulas. Often esophageal SEMS are placed for intractable benign esophageal strictures responding poorly to balloon dilatation [2]. Recurrent tumor in-growth following esophageal SEMS placement has been described leading to reemergent dysphagia and the same is usually managed with “stent in stent (coaxial stent placement)” procedures.

Despite the widespread use of esophageal SEMS placement for the numerous previously described indications, delayed prolapse and erosion of esophageal SEMS into the tracheobronchial tree are rarely reported [1, 3–6]. However, this uncommon complication can often present as a potentially life threatening emergency. We describe a patient with recurrent tumorous esophageal SEMS obstruction who developed this complication following third esophageal SEMS placement.

## 2. Case Report

A 66-year-old female presented with history of persistent cough and shortness of breath of 2-week duration. Cough used to worsen immediately on intake of liquids/solids and, for the same reason, patient complained of marked reduction in oral intake. There was no history of hemoptysis, wheezing, hoarseness of voice, or chest pain.

Patient had been diagnosed with advanced stage carcinoma of the esophagus, 15 months ago. At that time, patient had been evaluated for symptoms of dysphagia, loss of appetite, and significant weight loss. Upper gastrointestinal endoscopy examination (UGIE) performed at that time revealed circumferential growth at 25 cms from incisors and biopsy demonstrated moderately differentiated squamous cell carcinoma. Fine needle aspiration from a palpable right supraclavicular lymph node at that time also confirmed metastatic squamous cell carcinoma. Flexible bronchoscopy (FB) examination at that time was normal. In view of grade III dysphagia and metastatic disease, patient underwent covered esophageal SEMS (Hanaro stent; M.I. Tech, Seoul, Korea, 18 mm × 14 cm) placement in November 2012 and palliative chemoradiotherapy (cisplatin, capecitabine, and 30 Gy-(10 fractions), radiotherapy) was concurrently administered. For recurrence of dysphagia six months following esophageal stenting, UGIE was performed which demonstrated tumor in-growth for which a covered SEMS (Hanaro stent; M.I. Tech, Seoul, Korea, 18 mm × 11 cm) was placed inside the previous stent. Tumor in-growth recurred nine months later for which another covered esophageal SEMS was placed inside the two previous stents. Symptoms of cough and dyspnea occurred one month following the third esophageal SEMS placement and the patient was referred to the pulmonary outpatient clinic.

On examination, patient appeared emaciated. Pulse rate was 100/min, respiratory rate was 25/min, and rest of the vital signs and general physical examination were normal. On examination of the respiratory system, inspiratory crepitations were audible in bilateral lung bases. A clinical possibility of airway-esophageal fistula was considered and FB examination was performed.

**Figure 1:**
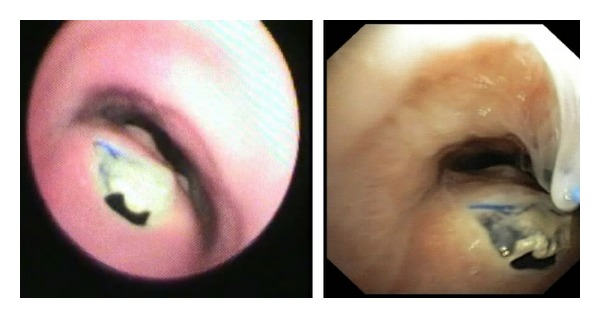
Flexible bronchoscopic image showing prolapse and erosion of the proximal end of esophageal stent through the posterior tracheal wall and formation of tracheoesophageal fistula (TEF).

FB demonstrated prolapse and erosion of the proximal end of esophageal stent through the posterior tracheal wall and formation of tracheoesophageal fistula (TEF) through which bubbling secretions was visualized. (Figure 1 and Video in Supplementary Material, available online at http://dx.doi.org/10.1155/2014/567582) On distal inspection, the wire mesh of the esophageal stent was seen over the posterior tracheal wall extending up to 1-2 cms above the carina. A diagnosis of tracheal erosion of the esophageal SEMS with TEF was confirmed. The patient was unfit for any surgical intervention and therefore bronchoscopic management was planned.

**Figure 2:**
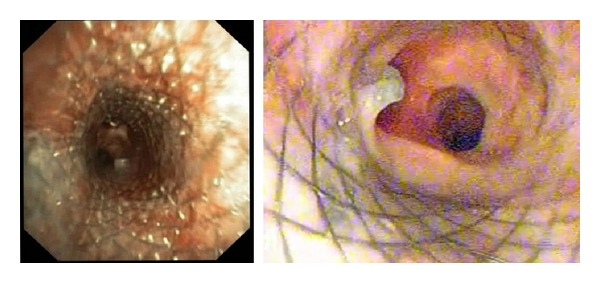
Flexible bronchoscopic image showing that the TEF and the eroded stent have been completely covered by the deployment of a covered tracheal SEMS.

Urgent bronchoscopic tracheal covered SEMS placement was planned. A covered tracheal SEMS (18 mm × 4 cms) was successfully deployed under flexible bronchoscopy and conscious sedation, to successfully cover the entire extent of the eroded esophageal SEMS and the TEF (Figure 2 and Video).

Patient had dramatic symptomatic improvement following tracheal stenting. Cough resolved, dyspnea improved, and patient was able to take oral diet without any symptoms of aspiration. On a follow-up flexible bronchoscopy examination performed two months following tracheal stenting, the stent was seen well in position. On last telephonic contact, patient was doing fine (no ongoing dysphagia since tracheal stenting was performed) and four months had passed since tracheal SEMS placement.

## 3. Discussion

Reported major complications associated with the placement of esophageal SEMS include significant bleeding, tumor obstruction, stent migration, airway-esophageal fistulization, gastroesophageal reflux, aspiration pneumonia, and persistent chest discomfort [4]. Esophageal SEMS placed into the upper third of the esophagus have been proposed to be more likely to develop complications due to close anatomic proximity to numerous structures [7]. The propensity for fistulization/erosion of the posterior tracheal wall occurs due to the fact that the posterior wall lacks cartilaginous support and is membranous in character. It has been proposed that stents placed in the aerodigestive tract may cause local inflammation and mucosal necrosis which make the walls more susceptible to perforation especially in situations of local presence of tumor or following radiation [3]. Our patient had received radiotherapy subsequent to the first esophageal stent placement. Also, due to the inherent structural geometric considerations of SEMS design, the bulbous stent ends which are thought to help anchor the stent to the wall by themselves lead to increased pressure stress at the stent ends (especially the proximal end in case of esophageal SEMS) increasing the chances of perforation [1].

Despite these factors, delayed tracheal erosion and TEF formation due to esophageal SEMS is a rarely described occurrence. However, erosion of esophageal SEMS into adjacent structures apart from the trachea such as aorta, bronchi, carotid arteries, and vertebral arteries has been described and vascular perforation is usually associated with a fatal outcome [1, 8]. Aggressive predeployment stricture dilatation and postdeployment balloon dilatation by an unskilled operator can increase the likelihood of these complications. No specific stent type can specifically be implicated in the causation of these complications. Like in our patient, mostly it is the upper edge of the stent which has been described to erode/prolapse in to the tracheal lumen.

The most important principle of management in the situation of tracheal erosion by esophageal SEMS is to stabilize the airway and to seal off the esophageal-respiratory communication. The same may involve a combination of or either of the following steps: esophageal stent removal and/or repeat esophageal stent placement/coaxial esophageal SEMS/tracheal covered SEMS placement/surgery in cases of benign disease [1]. Bronchoscopic approach to the placement of covered tracheal/bronchial SEMS placement may include either rigid or flexible bronchoscopy; however, rigid bronchoscopy is the preferred modality in situations with significant airway compromise [9].

A prophylactic approach for the prevention of this complications has also been suggested wherein parallel tracheal and esophageal stenting may be considered especially in situations where the large esophageal tumor burden increases the propensity of airway obstruction following esophageal SEMS deployment. In fact, in case a covered esophageal SEMS completely prolapses into trachea, fatal asphyxiation may occur which may not occur in case an uncovered stent had been utilized wherein the wire mesh can allow ventilation to continue [1].

The present case highlights the fact that, in rare circumstances, esophageal SEMS can penetrate into the tracheobronchial tree and present with emergent and often life threatening airway obstruction or aspiration. Occasionally, short of a frank rupture into the trachea, the stent can cause compression effect and tracheal lumen compromise also and sometimes a limited penetration by a stent wire has also been reported [10]. Endoscopists and bronchoscopists should be aware of this clinical entity and in patients wherein a high likelihood of airway complications is anticipated in view of large esophageal tumor burden; parallel tracheal and esophageal stenting should be considered.

## Supplementary Material

Video of the flexible bronchoscopy examination demonstrating the presence of eroded esophageal SEMS into the trachea. Covered metallic tracheal stent is being deployed using the flexible bronchoscope to cover the prolapsed esophageal SEMS.
